# “I’m not a graduate or doctor, yet we are all together:” articulating a partnership model for community-engaged research

**DOI:** 10.3389/fpubh.2026.1640624

**Published:** 2026-08-10

**Authors:** Jenna van Draanen, David L. Perlmutter, Jazmin Higuera Banos, Brenda Y. Goh, Cece Wettemann, Grover Will Williams, Nathan Holland, Esther Rourke, Rob Pitcher, Callan Elswick Fockele, Avery Park, Sierra Teadt, Thea Oliphant-Wells, Tessa Frohe

**Affiliations:** 1 Department of Health Systems and Population Health, School of Public Health, University of Washington, Seattle, WA, United States; 2 Department of Child, Family, and Population Health Nursing, University of Washington, Seattle, WA, United States; 3 Research with Expert Advisors on Drug Use, University of Washington, Seattle, WA, United States; 4 Department of Emergency Medicine, University of Washington, Seattle, WA, United States; 5 Public Health – Seattle & King County, Seattle, WA, United States; 6 Department of Psychiatry and Behavioral Sciences, University of Washington, Seattle, WA, United States

**Keywords:** community-engaged research, methods, peer research, research collaborative, substance use

## Abstract

**Introduction:**

Research with Expert Advisors on Drug Use (READU) is a community-engaged research collaborative of people with lived and living experience, clinicians, and academic researchers. We sought to evaluate the challenges and rewards of the partnership model.

**Materials and methods:**

Using the Peer Engagement Process Evaluation framework, the READU team collaborated on generating a list of focus group topics. All (*n* = 8) active team members participated in a focus group. Thematic analysis was used to identify key ideas.

**Results:**

Five main themes were identified. “Collaborative Research Model” relates to the importance of a shared set of values, power dynamics, and the importance of centering community in research. “Group Dynamics” describes occasionally getting side-tracked in the work, as well as closeness among team members. “Individual Growth and Skill Development” concerns personal and intellectual development, as well as the benefits and opportunities for bi-directional training. “Institutional Structure,” encompasses the logistics of a hybrid working model and compensation issues. “Moving Forward and Future Work” involves the process of ending a research project in a positive way and envisioning future opportunities.

**Discussion:**

READU’s partnership model is generally positive and mutually beneficial for all team members. All partners feel a sense of satisfaction and closeness, and that the relevance and impact of research is enhanced. Although some challenges have been identified, particularly related to compensation and the physical workplace, these are not intractable. The evaluation of this partnership should motivate researchers considering partnership with peer researchers and highlight practical considerations.

## Introduction

People who use drugs are increasingly engaged in developing health programs and policy (1, 2) and in research that informs programming and policy (3–5). Excluding people who use drugs from research on and about them has ethical and scientific ramifications, leaving community members feeling disrespected, stigmatized, and exploited (6), sometimes leading to de-stabilization and further marginalization (7). Moreover, research conducted without input from people who use drugs may not be relevant to their interests and diminish trust in academic institutions (3).

Community-engaged research (CEnR), a framework for the inclusion of community members or peers, enhances rigor and relevance of research, as well as timeliness, accessibility, and actionability of results (1, 8). While research questions and designs are typically generated from the top down, CEnR can involve peers from the beginning as equal partners (9). Community partnerships are collaborative, valuing unique strengths and responsibilities of community, and frames community as a facet of identity (9).

Roche and colleagues define three practice models for engaging people who use drugs in research: (1) advisory roles without much decision-making power; (2) being hired as staff to perform discrete, pre-determined tasks such as data collection; (3) participation in shaping the research question, design, dissemination, and potentially sharing ownership of the data (10). Prior research collaboratives with people who use drugs have shown that partnership enriches the process for all parties and enhances the quality of the research (11–13). CEnR collaboratives recognizes lived and living experience of drug use as an essential form of expertise that complements scientific expertise, actively engaging peer researchers as equitable partners throughout the research process. Peer researchers may contribute experiential knowledge, trusted community relationships, and insights regarding the drug use landscape, while academically trained researchers may contribute theoretical, methodological, and technical expertise. Through a shared purpose and commitment to promote the health of people who use drugs, peer and academic partnerships can foster mutual learning, shared decision-making, and the co-creation of knowledge that is rigorous, ethical, and responsive to community needs.

Inclusion of people who use drugs on a research team, however, does not guarantee authentic or meaningful interaction. Unexamined power dynamics and a failure to meaningfully involve people who use drugs in study planning breed disappointment and feelings of exclusion (3, 14). This underscores the need to identify engagement strategies that are authentic and mutually beneficial.

Existing literature focuses on the ethical issues and engagement models for people who use drugs employed as peers in service delivery settings (14, 15), and employment of members of other marginalized communities as peer research assistants or associates (14–16). Less is known about how to form research partnerships that are respectful, equitable, and responsive to the interests of people who use drugs and which CEnR models are most effective. In this article, we describe the formation of Research with Expert Advisors on Drug Use (READU), an active CEnR partnership of peers with lived and living experience of substance use, academic researchers, and practitioners. We address the following questions in the context of the group’s contribution to several research studies:

- What are the benefits and challenges of this research partnership for READU members?
- How do READU members want this partnership to evolve?

## Materials and methods

### Formation of READU

Two participatory research projects were created to respond to local evidence needs regarding overdose response within first response systems (e.g., emergency medical services, firefighters). These studies focused on adaptation of harm reduction and other overdose prevention-related interventions from conventional clinical settings into prehospital care. Results of these studies have been previously described elsewhere (17, 18). These projects were launched in partnership with community organizers, local public health agencies, harm reduction service providers, academic researchers, and clinicians seeking to engage people who use drugs to inform the development of an opioid overdose prevention program. The two qualitative pilot projects were designed to have ongoing involvement of peers with lived and living experience of substance use as paid team members. Because funding for the initial two studies supported recruitment and hiring of peer researchers, their involvement in these early projects was more pre-defined than the READU model would evolve to be. This also meant that the research questions for the two initial studies were defined in advance of recruitment. This stepwise evolution allowed the team to build consensus around norms and develop crucial skills for conducting research before shifting to a greater degree of collaboration, including a process for assessing whether potential research questions and proposals are consistent with READU’s guiding values. More details on the partnership model can be found in Supplementary material 1, and the team agreements can be found in Supplementary material 2.

Recruitment for READU occurred between September–October 2021. First, the peer researcher position description was drafted outlining, in lay language, both studies and qualifications for the position including: (1) the desire to meet the needs of people who use drugs; (2) experience working on a team; (3) comfort with reading, writing, technology, and sharing thoughts in a group setting. It stated that no research experience was required, and researchers would receive training. The description specified the range of hours a peer researcher could expect to work each week, the hourly pay, the pay schedule, the expected duration of the position, and all the questions that research staff would ask in an interview. The position description was intended to convey an accessible and flexible environment where fit would be mutually evaluated after 3 months. In addition to hourly wages, peer researchers received a weekly stipend meant to help with transportation, technology access, and/or food expenses. The stipend was intended to address any barriers that might prevent meaningful participation. This is an acknowledgement of the costs associated with work, particularly for individuals who are otherwise socially or economically marginalized.

Five peer researchers joined five academically-affiliated researchers/staff in November 2021 to form READU. Four of the peer researchers were recruited through partnerships the academically-affiliated researchers had established with community-based service or advocacy organization. At least one of the peer researchers had prior experience serving on a community advisory board to inform research and another had experience as a community organizer. These peer researchers had a variety of experiences in terms of substance use history, prior engagement with emergency medical services and the criminal-legal system, and differed in terms of active substance use, harm reduction practice, and abstinence. When fit was mutually assessed after 3 months, two peer researchers left the team: one due to time constraints and another due to difficulty upholding team agreements. Bi-directional training throughout the partnership included academically-affiliated researchers sharing knowledge about qualitative methods, institutional and community-driven research ethics, sampling, and interviewing skills, and peer researchers sharing knowledge about the context surrounding local overdose response. During this period, the team also discussed peer researchers’ preferences for specific roles in the overdose response study (e.g., interest in conducting interviews) as well as modifiable elements of the study design (e.g., selection of sites for data collection). The team met weekly, initially for bi-directional training and team building; later, for study planning activities like co-development of interview protocol and data collection strategies. When the two pilot studies began, all but one peer researcher participated in conducting interviews. All peer researchers participated in the analysis process. Likewise, all academic researchers participated in data collection and analysis. Peer researchers participated in all phases of research and ultimately presented findings in conferences, manuscripts (17–19), and community-designed knowledge products (e.g., infographics, zines) (20). Research activities that peer researchers were generally not involved in were the administrative, for instance, logistics of weekly meetings, interfacing with the university, coordinating with the service providing organizations which were the host sites.

Due to the projects’ funding, some peer researchers could not be formally employed by the university and instead were affiliated by partnership. As a result, keycard access to university buildings where most team meetings occurred was not permitted due to departmental policy. Peer researchers instead had to meet at the entrance and walk upstairs with an academic researcher. Peer researchers were paid either weekly in cash for those without bank accounts and/or via check for those who were able to be established as contractors or employees with the university. After the pilot projects’ data collection and analyses were complete, the present study sought to examine the READU partnership benefits, challenges, and hopes for future evolution with the eight remaining team members.

### Conceptual framework and data collection

The Peer Engagement Process Evaluation Framework (21) was used to identify relevant constructs to guide the focus group discussion, including compensation-related issues, creating a supportive working environment, power and identity negotiations in a new work setting, capacity building, and shared goals for the future. The READU team collaboratively decided to focus on these constructs; at a weekly meeting, a research assistant described the framework to the team and led a discussion to identify what constructs and issues were most salient to the group. Resulting focus group questions can be found in Supplementary material 3. READU team members discussed these and produced a list of discussion topics. One team member formatted these topics into focus group questions, which were subsequently reviewed and revised by the entire team. A 120-min focus group was conducted with all (*n* = 8) active READU team members. This included four peer researchers, three academic researchers, and one graduate research assistant. Peer researchers and the research assistant were compensated at their regular hourly wages for participating, and the focus group was scheduled into their typical schedule. Academic researchers elected to join voluntarily. The focus group was conducted in a hybrid- video conference and in-person format. Video conference was facilitated by Microsoft Teams. The hybrid format was in keeping with READU’s values around flexibility for team members and accommodated team members who were unable to be present in person. It was facilitated by a graduate student who had previously worked with READU and had formal training in qualitative methods as well as practical experience (ST). It was audio-recorded and transcribed verbatim using a professional transcription service. In order to accommodate any team member who might be uncomfortable or unwilling to share thoughts in a group setting or share thoughts that might be critical of the group or an individual, focus group participants were invited to share thoughts privately with the facilitator by email/phone/text at the beginning of the focus group, though none did. Written informed consent was obtained by all participants. For those participating remotely, consent was obtained by email. Only focus group participants and the facilitator were present.

### Data analysis

Analysis utilized a thematic analysis approach (22). Two undergraduate student coders, who had no prior experience working with READU, reviewed the transcript and created initial impressions for codes. One READU supervisor, who did not participate in the focus group, and the two coders then engaged in a workshop with the whole READU team to collaboratively develop a codebook using the notes and through discussion of the transcripts. Two student coders used the final codebook to independently code all transcripts during the primary coding phase, followed by a secondary coding phase in which the other student coder and the READU supervisor independently coded all transcripts. Any inconsistencies or disagreements were discussed regularly among the coders, documented, and resolved by the READU supervisor. The codebook did not have a hierarchical structure (e.g., “parent” and “child” codes). The student coders reviewed the completed codes and associated data, subsequently organizing them into candidate themes and sub-themes. Dedoose was used for coding and codebook organization. Themes were referenced against the larger dataset for coherence, formally defined, and named. Preliminary themes were discussed with the READU team at this point, which informed how they were ultimately defined in the manuscript.

## Results

### Theme 1: collaborative research model

The READU team reflected on three main elements of their collaborative research model, illustrated in subthemes below, including: (1) teamwork and a shared sense of values; (2) leadership, power structure, and hierarchy; and (3) community focus, interaction, and engagement. Illustrative quotes are captured in Table 1.

**Table 1:** Summary of thematic analysis findings.

Theme	Subtheme	Representative quotes
Collaborative research model	Teamwork and a shared sense of values	“This project feels different from other research I have done because I feel like I’m part of a team with a shared goal of making a real impact.” “I think that it’s really held strongly to the core beliefs that were created by the group, which is also a really unique aspect. I think sometimes groups will come up with their mission statement independently and then kind of find people to come in and fit within that mission, whereas this was really like, Here’s our group of folks. How do we want to orient this and to experience the projects that we are going into together.” “I’m grateful for the project’s commitment to dismantling power structures and creating a truly collaborative and collegial environment.”
	Leadership, power structure, hierarchy	“And just to add with that, that’s something also I’ve appreciated too, just because we have three doctors on the team and college graduates, just because I’m not a graduate or a doctor yet we are all together. There’s no differential stages or steps or positions.” “So it’s like you never come fully yourself, but I think the way you have it, we have it, you can just come yourself, at all aspects” “Yeah, or like figuring out how to behave in the right way under certain circumstances with certain kinds of people. I think that and what I mean is I think that I am a different person when I’m interacting one-on-one with you than when I’m interacting with one-on-one with [*team lead*] or as then as a group…”
	Community focus, interaction and engagement	“it was a perspective expanding experience to hear from people who use drugs” “including more people with lived experience […] would be better if possible “this just feels like the most authentic way that it’s been done, at least in my experience before.” “I think this has been a really good example of how successful community work can be. And how it can be structured in a way that really is equitable and it gives everyone equal opportunities just to talk and like have their voices heard.” “that’s exactly what this group is designed for, was to hear our opinions and what we have to say.” “and the hope and aspiration is that the work that we are gonna do is going to actually have impact or policy change or programmatic change or something like that, which is something I’ve never felt before. So for all those reasons I’ve just been really appreciative of this time.”
Group dynamics	The duality of getting side-tracked	“There may have been times where I felt we were slightly drawn off track. Or we could have used… It would’ve been helpful to retain some focus at times. But that I think got dealt with. We worked around it for the most part.” “Yeah, I know what you mean. We kind of took some tangents, especially earlier in our meetings together, I think…” “It’s been really flexible and really kind of permissive as far as what we do when we get together to meet or when we do, yeah, any meeting I think that there’s an agenda that’s like, it’s present but we do get side-tracked a lot and I think it’s been helpful for getting to know each other, and I think we have learned other things that way…”
	Sense of closeness	“I’ve never been working so closely with people on a project, meeting so many times a week and really doing everything together, like every step of it, all as a group.” “I also just really have appreciated the authenticity of the group of meeting people where they are at and really being holistically caring for the whole person and not just the work.” “I connect with people around when we break out of our assigned sort of work roles, and when we are interacting with one another outside of wearing very sort of tightly assigned hats and doing these specific tasks. And that helps me feel like I have a sense of people that I’ve spent a lot of time working with as both who I interact with as well as all the other lives that they have lived. And I feel like I get a way better sense of who of a person and that helps me work with them better. And I feel like that gives me an opportunity to bring parts of myself that I do not feel comfortable bringing to a normal working environment. And I think that improves working dynamics quite a bit.” “I too have been a part of many different research projects over the years, also trying to incorporate people with lived experience and this just feels like the most authentic way that it’s been done, at least in my experience before.”
Individual growth and skill development	Intellectual and personal growth	“Well, I also have been loving it, I feel like my touch points have been more in the clinical setting, so feeling like… I think more of in a much more sterile way and thinking about programs and policies and protocols that govern my life at the hospital. And I feel like this has been a really amazing opportunity to just kind of just really listen and feel guided and ask questions about my current clinical setting in a way that I never would have otherwise. “” “One thing I could take away from here is I’ve worked on my social anxieties.” “And I feel like I’ve learned a lot from everybody who’s been participating, and I think that I’ve also learned a lot about just how research works, I think that a lot of what has been really valuable about the process for me has just been getting the chance to learn a lot of stuff that I would not otherwise just about how research works and yeah, it’s also it’s been a fun and good time generally.”
	Training and opportunities for bi-directional learning	“…I felt like that application was really helpful for what you were saying…Cause it was like this was like an exercise we did and then when we actually did it for real, it was like, ‘Oh, we kind of did this before.’” “…I think probably… I’m definitely more of a learn-by-doing person, so that stuff was really helpful to get me in the mindset to get involved with the research.”
Workplace structure	Coming to work	“I do not think that I would’ve really been able to participate meaningfully if it had not been for the remote work being possible. So I thought that was really great. And also it was awesome to be able to also meet in person.” “Security situation sucked ‘cause I always felt like I was inconveniencing [chuckle] someone or like, and partially it was because I was usually not early. So I can own that but yeah it just, yeah it was kinda hard ‘cause you could not just get right in, upstairs anyway.” “I do not think that I would’ve really been able to participate meaningfully if it had not been for the remote work being possible. So I thought that was really great. And also it was awesome to be able to also meet in person.” “Security situation sucked ‘cause I always felt like I was inconveniencing [chuckle] someone or like, and partially it was because I was usually not early. So I can own that but yeah it just, yeah it was kinda hard ‘cause you could not just get right in, upstairs anyway.”
	Getting paid to work	“This question just made me think about sustainability and I feel like part of [equity] has been paying people for their experience and…as a way of saying, ‘I respect your experience and you are like a full member of this team’”
Moving forward and future work	Ending in a positive way	“I feel like it was a valuable experience regardless of like, once it’s over I’ll be happy that I participated in it” “I do not see any other way to introduce or prepare us for the maybe inevitable end because we already came into this situation knowing that there was, that this wasn’t forever… “I’m adamant ‘cause I think that us doing this is gonna create more, so I do not think we are done, is basically what I mean.” “I think maybe talking about like how people can stay involved in research or in policy making decisions or stuff that would inform policy making decisions.”
	Future directions	“I’m really interested in things related to overdose prevention, overdose prevention sites, and I think I mentioned a long time ago like Good Samaritan and drug homicide laws.” “It seems so cool to think about this as like planting a seed for like…doing research that’s on topics that are actually generated by the communities that are affecting them, rather than just sort of participating in topics that we have decided on in advance.” “…I really do feel like the way that this team has been able to kind of reach out to others and talk about what we have been doing and really integrate itself both into the systems that we are trying to work within, but also just within colleagues and other people. I just think it’s been all around really successful. And maybe I’m being like super hopeful. There aren’t always projects that I feel this way about. Like sometimes you, at least in my world, sometimes you run a study, write a paper, and you are like, well that’s that, and you kind of like, move on. But this one totally does not feel like that.”

#### Teamwork, experience, and a shared sense of values

When describing their teamwork and a shared sense of values, participants discussed collaboration and what it meant to each of them in their personal and collective identities to be part of the CEnR team. Participants expressed appreciation for being part of a team that felt more collaborative, collegial, and mission-driven than other projects they had participated in. They liked being part of a team with shared goals of policy impact and reducing stigma, specifically elaborating on the meaning and importance they felt as members of a team working on campus conducting research on harm reduction. Participants discussed creating their mission statement together and articulated shared movement toward a collective goal that had the potential to make change in the community. Participants expressed gratitude for their team’s shared commitment to dismantling power structures (further elaborated on in the “group dynamics” theme below), which they described as part of their collective identity. Participants were appreciative of the space created within the team to conduct particularly meaningful work based on common values and goals. Several participants referenced the ethics and core beliefs uniting the team, giving them a shared sense of purpose.

#### Leadership, power structure, hierarchy

This subtheme related to a shared decision-making process where people with different backgrounds, professions, and experiences could work together. The leadership model was described using words like “flexible,” where there was not an imposed way of doing things and everyone’s opinion was valued. Personal and professional identities came up and participants described both comfort and discomfort with this process. Ultimately, participants spoke about this as part of learning how to interact with each other and work together in an environment with less barriers.

#### Community focus, interaction, and engagement

Participants were positive about their research involvement and emphasized the value of meaningful involvement of people with lived experience in every stage. They felt that this was an authentic way to conduct research, describing the importance of providing equitable opportunities for all voices to be heard, and noting how this can lead to practical changes in policy and intervention strategies in community. Although the participants generally viewed READU as a successful example of engagement and appreciated how the team connected people across a variety of professional, research, and community roles, they expressed a desire to include more people with lived experience.

### Theme 2: group dynamics

Within the over-arching theme of group dynamics and team functionality, subthemes of getting side-tracked and a sense of closeness emerged.

#### The duality of getting side-tracked

Participants discussed how sometimes during team meetings, discussions would lead to tangents which felt like a lack of focus. Some participants described how the discussion’s flexibility and permissive aspect may lead to digressions, but helped them get to know and learn from one another better than highly-structured meetings. On the other hand, one participant expressed frustrations with the tendency to be side-tracked due to prior experience working in more structured, task-based settings.

#### Sense of closeness

Participants described a personal connection with the people they work with, liking other team members, caring for each other, and enjoying time together, which they felt was beneficial to the group dynamic and created genuine relationships within the team.

### Theme 3: individual growth and skill development

Participants discussed personal growth and development from READU participation. Subthemes related to intellectual and personal growth as well as opportunities for bi-directional learning and other training.

#### Intellectual and personal growth

Participants shared a variety of ways in which they had grown individually and as a team. Participants explained learning new things about research methods, substance use policy, and clinical interventions. They also gave examples about how they had grown personally (e.g., decreased social anxiety) and professionally (e.g., exposure to a different setting and new type of innovative research). Many participants appreciated the flexibility and openness of the working structure (e.g., having a variety of roles, responsibilities, and participation options that could be aligned with one’s skills and desires) which facilitated team growth.

#### Training and opportunities for bi-directional learning

The initial weeks of READU were devoted to providing broad, didactic training around conducting research, with a focus on qualitative methods. Some peer researchers noted that including high-level concepts (e.g., “What is research?,” “What are research ethics?”) before methods was useful. No peer researchers had prior exposure to research methodology, and they expressed that the training had adequately prepared them. Peer researchers appreciated the opportunity to apply concepts from the training via interactive exercises, including mock interviews to practice data collection and creating a codebook together. The emphasis on discussion and taking time to evaluate the “why” of research practices was seen as an important element of rigorous training. Participants without lived experience expressed a desire to increase bi-directionality, in line with the original intent, where peer researchers could also offer training on relevant topics (e.g., challenges with probation, permanent supportive housing structures, drug market fluctuations, etc.).

### Theme 4: institutional structure

Participants discussed the logistics of a hybrid working model and compensation issues, with these subthemes making up an overarching institutional structure theme.

#### Hybrid model

Participants noted several benefits and challenges to participation. The flexibility to work remotely increased accessibility and convenience while reducing anxiety about missing work due to extenuating circumstances. However, peer researchers noted challenges using technologies required for remote participation and sometimes lacking access to the right equipment. Some team members found it difficult to keep the technology in good working condition, while others felt that remote data collection with two interviewers was challenging, particularly navigating between an interview guide and the video conference on a single screen.

For most team members, in-person meetings on the university campus provided benefits and challenges. Team members enjoyed the building’s amenities, namely new technology that made hybrid collaboration easier, as well as access to free refreshments. However, an institutional work setting was a strain for some. Peer researchers felt frustrated about their limited access. Both peer researchers and academic researchers worried about the risk of unwanted encounters with campus security, and alternatives were tried, including meeting on the first floor where no keycard was required. Unfortunately, this was challenging for some members because it was less private, with fewer amenities. A suggestion for future work included holding in-person activities somewhere “that is low-key, not necessarily in an institution of some kind,” but this would mean losing the benefits that came with the institutional setting. After weighing the risks and benefits, the group decided to remain in the high-barrier space.

#### Getting paid for time

For some team members who were trying to reduce or eliminate substance use, payment in cash was occasionally challenging. One peer researcher noted the risk associated with “having a decently large amount of cash all at once right before the weekend,” and participants discussed potential solutions, like payments earlier in the week or an intentionally deferred payment, both of which were subsequently implemented individually when requested. Despite these concerns, all participants agreed that compensating people with lived and living experience for their expertise and knowledge was a shared value. Because compensation was a way to recognize peer researchers as equal partners on the projects, participants felt that continuing and sustainable funding streams were critical.

### Theme 5: moving forward and future work

Discussions about sustainability, next steps, and future work elicited two distinct subthemes: ending in a positive way and future directions.

#### Ending in a positive way

Participants reflected on ending the projects in a positive way, finding ways to stay involved in research, policy development, and communicating research findings as part of their community responsibility. While there was overarching uncertainty about next steps for the research, most believed that their work would “speak for itself” and would ultimately lead to future opportunities. The participants observed that academic institutions could lower barriers for community engagement and community-driven research and hoped that their research would not only impact local overdose response but also change the way community research is done. They noted structural agreements that would ensure future READU work would grow out of the group’s shared values (see Table 1) and hoped the same could be true for other teams.

#### Future directions

Participants expressed interest in future research on overdose prevention and harm reduction sites, as well as other topics they were passionate about. They highlighted the need to structure community work equitably, ensuring everyone’s voices are heard and data should inform policy changes. Finally, all participants hoped to champion involving more people with lived experience in substance use research.

## Discussion

We conducted a qualitative study to understand the benefits, challenges, and desired evolution of a research partnership with academic and peer researchers focused on substance use. Themes that emerged from the focus group included reflections on the collaborative research model, group dynamics and team functionality, experiences of personal growth and development, institutional structure, and future work. Participants’ emphasis on power sharing, trust and relationship building, fair compensation and recognition, flexibility and accessibility, and reciprocity were likely shaped by the longstanding stigmatization, marginalization, and exclusion of people who use drugs from substance use research. Their reflections of the READU model of collaboration suggest that it started as a partially *contractual* model (23), but eventually evolved into a *co-created* model, where professional researchers and community members partner together at all stages of the scientific inquiry process, co-designing research questions driven by community needs, co-learning, and co-creating knowledge (24–32). Additional examples regarding the application of READU’s model of collaboration across the partnership and planning, methods, results, and sustainment research phases, have been reported elsewhere (33).

There were many articulated benefits and positive elements of the collaboration model. Notably, READU’s model and structure elicited a strong sense of “togetherness” with shared values, teamwork, and closeness described by all. Like other CEnR studies, READU members valued regular meetings and the opportunity to learn from each other in a safe and supportive environment (34–36), emphasizing that these interactions strengthened teamwork and enhanced success (37). Not many published CEnR projects describe efforts taken to promote partnership development between professional researchers and peer researchers (38). Those that do, have described their structured team-based co-learning activities as intensive sharing of experiences and stories with listening, knowledge acquisition, and relationship building as pillars of this work; this resembles an experiential learning style, “reflection-action cycle of learning and application,” (36, 38–42) similar to READU’s bidirectional training model.

READU members expressed a strong feeling of identification with the team but also felt like they had room and support for their own personal growth and development. Similar to the theme of personal growth and development identified in this study, peers engaged in other research collaboratives have reported increased self-confidence through mentored capacity building in a highly collaborative working environment (15). Members of our team appreciated flexibility in how individual members treated each other and how team processes adapted to the preferences and circumstances of individual members.

Participants in the focus group described several challenges. First, the group often became side-tracked during team meetings, which was especially challenging for those with prior experience working in settings requiring relatively more intensive focus and output. READU has partly addressed this by providing opportunities for research activities with varying levels of structure, for instance, discrete data collection or logistical tasks rather than less structured group brainstorming activities. Others brought up discomfort with wearing both personal and professional “hats” in the same space, an idea described by other CEnR teams and researchers (43).

Logistical challenges periodically arose for peer researchers who occasionally participated remotely, and these barriers persisted despite efforts to mitigate them. READU has remained committed to working with peer researchers to ensure they can continue to participate to their desired extent, whether through weekly troubleshooting of technical issues or facilitating transportation. Imperfect circumstances with both the in-person meeting space and compensation model led to challenges for peer researchers. These types of logistical challenges have been previously identified in CEnR projects and can increase occupational burnout, compassion fatigue, job cynicism, and high turnover (44). While the CEnR literature commonly cites occupational hazards, psychological stressors and the feeling of tokenization of peer researchers (43) especially when they are not given meaningful work and provided space to give input on substantive research issues, these challenges were notably absent from our focus group discussion. Academic researchers were conscientious of these risks from the outset and implemented strategies to address them from the outset. READU has prioritized flexibility, for instance, through the provision of remote work technology, provision of stipends to accommodate the cost of working that might detract from hourly wages, and the ability for peer researchers to have choose what parts of the research process they would like to be involved in. This underscores the importance of creating a variety of different options through which to contribute to the generation and synthesis of knowledge, as the READU model did, in contrast to other CEnR projects that assign discrete tasks or ask for feedback on previously completed work (45, 46).

While specific changes to the model were not suggested, participants shared thoughts about sustainability, next steps, and future work. They hoped future work would be driven by the scientific interests and social engagement of all team members. The team had no guaranteed funding for future work when this study was conducted; however, the team has since secured additional funding and participated in several new projects driven by their diverse interests (47). Projects have been decided on through a group consensus model, and the team has developed a system for evaluating new research opportunities and creating contracts to ensure mutual understanding before embarking on new research or advisory projects. Refer to Supplementary material 4 for more details on criteria used to evaluate project opportunities.

### Limitations

This study was limited by a relatively small sample size, single focus group design, and social desirability bias. Two team members had left the team prior to data collection and were not able to participate in the focus group, so it is likely that their perspectives differed from those who did participate. Negative or contradictory opinions may have been especially difficult for group members to share, although confidential and private opportunities for submitting thoughts outside of the focus group were provided. Due to the highly contextual nature of the situation and CEnR model, results from this focus group may not be generalizable. Nevertheless, in the spirit of continually improving CEnR, we offer our experiences and partnership model for others to learn from, critique, and build on.

### Strengths

The present article makes several contributions to the existing literature. Much of the research concerning people who use drugs’ involvement in CEnR has been generated from projects well into their development or after their completion. We offer the perspectives of a research collaborative at a critical, early stage of relationship-building, to understand what has worked well and what should improve. We build on existing knowledge about engaging people who use drugs as partners through our practical experience. And crucially, we identify priorities as we co-create a research agenda driven equitably by the interests and contributions of all team members.

## Conclusion

Partnering with people with lived and living experience has the potential to enhance the quality of research. We offer our experiences as a research collaborative to demonstrate the challenges and rewards of CEnR, and highlight practical considerations for researchers of working with people who use drugs. READU members valued the opportunity to develop closeness as a team, develop personally and professionally, and to learn from one another. Adjusting to the pace of research and staying on track, as well as practical considerations related to compensation and creating an accessible workplace, were identified as challenges. As READU has matured, it has evolved to a collaborative that is increasingly co-created, with members working together to generate new research ideas and pursuing opportunities for collaboration.

## Data Availability

The datasets presented in this article are not readily available because due to the identifiable nature of qualitative data, raw data is not available. Requests to access the datasets should be directed to jvandraa@uw.edu.
