# Supplementary material for: “I’m not a graduate or doctor, yet we are all together:” articulating a partnership model for community-engaged research
Source: Front Public Health. 2026 Aug 10;14:1640624. doi: 10.3389/fpubh.2026.1640624 (PMC13500621; doi:10.3389/fpubh.2026.1640624)
Supplement: Supplementary file 1 [file Table_1.DOCX]

Supplementary Material 1. Research with Expert Advisors on Drug Use (READU) partnership model description

| **Community Partnered**  **Research Checklist Questions^1^** | **Description of READU’s Model** |
| --- | --- |
| Partnership and Planning Phase |  |
| 1. Does the study address how professional researchers and community co-researchers initiated and engaged in partnership development?  2. Does the study discuss how professional researchers and community co-researchers foster an equitable, team-based relationship?  3. Does the study clearly state how the aims of the research address an important issue identified by the community of interest? | Previously, partnerships and projects were initiated actively by the READU team and passively by external academic researchers or stakeholders, who approached the READU team with existing projects. Grant requirements and READU’s involvement in existing projects that external stakeholders initiated, limited the scope and direction of the research. Currently, the READU team is initiating original projects and diversifying grant funding applications for these projects to ensure that the partnerships and projects are mutually driven as opposed to researcher driven. Issues and priorities are now identified by community co-researchers and project ideas and research questions are developed by the READU team collaborative. READU members collectively decide about READU’s involvement in prospective projects that are suggested by external stakeholders, partners, and funders through a democratic and collaborative process. Existing projects that are proposed by external stakeholders are screened by the whole READU team to determine if projects are meaningful, consistent with the mission of the team, and beneficial to the larger community. The READU team has ongoing discussions regarding the ethicality of the research and the research’s potential for harm to the community. Potential partners must also sign a partnership contract to ensure accountability and transparency in the partnership. The READU team regularly engages in bi-directional training where community co-researchers share their lived and living experiences, while formally trained researchers share knowledge about research methods. To acknowledge community co-researchers' contributions and protect their intellectual property, community co-researchers are credited and included as authors in all publications and presentations, with their consent. |
| Methods Phase |  |
| 4. Does the study design align with the nature of the research question(s) and meet the needs of the community of interest?  5. Does the study use a recruitment strategy appropriate to the research question(s) and respect the community of interest’s culture, values, and beliefs?  6. Does the study collect data in a way that answers the research question(s) and respectfully follows the cultural nuances of verbal and non-verbal communication practiced by the community of interest?  7. Does the study provide community co-researchers with adequate resources and strategies for working with and collecting data from the community of interest?  8. Does the study facilitate activities to promote inclusion and a sense of belonging for professional researchers, community co-researchers, and the community of interest? | The READU team has primarily conducted research using qualitative methods, always choosing the most appropriate methods for the research question being asked and the population/problem being studied. The qualitative methods used have allowed the team to have meaningful engagement and interaction in community settings, giving study participants (often people who use drugs) with the opportunity to share their stories. Inclusion and exclusion criteria are chosen to fit each research question, and consideration is given to maximizing representation from participants whose perspectives have historically been excluded from research. The privacy and confidentiality of participants are protected throughout the course of the study. In addition to the secure storage of participants’ identifying information and the de-identification of study results, participants may elect to not disclose any information or opt to redact any information provided. To ensure that participants feel comfortable when participating in the research, the READU team has ongoing discussions regarding people who use drugs’ safety and the potential stressors that they may experience. Resources and equitable compensation are provided to participants for their time and participation. Oftentimes, an academic researcher is paired with a community co-researcher during data collection to increase participants’ comfort levels. Trauma-informed language and approaches are used and data collection tools undergo extensive piloting and revision beforehand to prioritize the well-being of people who use drugs. Regular team meetings and bi-directional training are conducted to debrief data collection activities, address issues that arose, and provide additional support for team members. READU members are always provided with alternative ways to participate in research activities that do not involve direction interaction with participants or spaces that might increase risk or discomfort for them. For example, when collecting data at harm reduction social service locations, READU team members may opt to stay in the research office and do office-based tasks (e.g., help design recruitment materials, etc.) so that they do not need to choose between activities that may make them uncomfortable (data collection) and earning money. Through team-building exercises and social events, community co-researchers and academic researchers build comradery, learn from one another, and grow together as a team. Members of the READU team uplift and support one another by reflecting on the work and celebrating each milestone. |
| Results Phase |  |
| 9. Does the study discuss how professional researchers and community co-researchers collaborated in analyzing and interpreting research data and findings? | Formally trained academic researchers and community co-researchers on the READU team collaboratively analyze and interpret the research data and findings. Community co-researchers are presented with various opportunities to participate in data analysis and make decisions regarding the extent of their involvement. Bi-directional training on the analysis process and steps are provided by academic researchers frequently, with practice sessions built into team meetings. The READU team begins the analysis process by familiarizing themselves with the data and engaging in group analysis. To support READU members, examples of how to analyze the data are provided and additional assistance is offered. Community co-researchers can request individual sessions with academic researchers to seek clarification, complete the step of the analysis together, and ask for additional support or resources throughout the analysis phase. Generally, READU members are assigned to review each other’s work to establish reliability and accuracy. Whenever possible, the READU team intentionally builds additional time into the study timeline to allow for greater flexibility and to ensure that community co-researchers can learn and work in conducive environments. After written findings have been drafted by academic researchers, the preliminary findings are brought back to the READU team for collective interpretation to ensure that the findings are consistent with the data that has been collected. The READU team together contextualizes and discusses the meaning of these findings and their broader implications for the research. |
| Sustainment Phase |  |
| 10. Does the study consider various methods of dissemination to reach non-academic audiences and benefit the community of interest?  11. Does the study translate into a change in policy, practice, and/or other “real-world” actions? | The dissemination of READU’s research to both academic and non-academic audiences is achieved through a variety of methods and channels to facilitate knowledge sharing. The READU team collaboratively identifies the intended audiences and channels of dissemination at regular team meetings. A dissemination plan is created and iteratively revised based on these ongoing conversations. Community co-researchers provide important insights for the use of lay language, accessible communication, social media channels, and print media channels when disseminating information to members of the community. Academic researchers provide suggestions for the dissemination of information to academic audiences through scientific publications, academic conferences, and broadcast media channels. The READU team constantly prioritizes knowledge sharing with the study participants and the community and seeks to disseminate the findings of the research through creative channels and methods, such as art or storytelling. Members of the READU team are directly involved in developing messages and tailoring the content to the intended audiences to increase the accessibility and relevancy of the information. All team members (academic and community co-researchers) who elect to participate are involved in public presentations to share study findings. At the core of READU’s work is the desire to improve the health and well-being of people who use drugs and their communities. READU’s research has led to the development of policy and practice recommendations which are being translated into actionable change in the community. Recommendations are informed by the data collected from members of the community and are co-created by academic researchers and community co-researchers. |
| ^1^Notes: The description of READU’s partnership model was guided by the prompts in the community-partnered research appraisal checklist (Park & van Draanen; *under review*) | |

Supplementary Material 2. Team agreements

| **Our vision** | **How we get there** |
| --- | --- |
| Trusting each other | 1. Assume positive intent in our words and actions 2. Clear and open communication |
| Everyone feeling safe and comfortable | 1. Respecting other people’s point of view and respecting differences 2. Being flexible, understanding when things don’t go as planned or when things need to change 3. Being patient with ourselves and each other 4. Keeping what is said in our meetings and in our work confidential 5. Respecting people’s privacy, respecting what people choose to share and what people choose not to share with others 6. Respecting boundaries: watch what you talk about and be aware of the potential that other people might not be comfortable with the same things as you 7. Personal safety: physical fighting is not tolerated |
| Commitment and engagement from all team members | 8. Bringing our best selves to the projects, holding ourselves to a high standard of professionalism  - Being on time - Staying focused - Taking our roles seriously - Let team know if you can’t make it to a meeting - We don’t work evenings and weekends: if you’re trying to get in touch outside of business hours, you may not get a response until the next day/workday |
| Clear roles, responsibilities, communication, and feedback | 9. Reviewing processes in detail for each new phase of the project 10. Assigning responsibilities before starting a new phase of the project 11. Meeting with team leaders individually every 3 months to check in 12. Managing expectations of each other, being understanding of other people’s needs |

Supplementary Material 3. Focus group questions

| Reflections on Collaboration and Group Experience | - How has the process of working together been for you personally? - How do you feel the experience of working in this group has prepared you for future professional or research opportunities? - Do you feel that your experience and perspective were respected by others in the group? If not, what could be done to help you feel more heard? - What could our team do to support individuals in bringing more of themselves into the space? - How do you feel about the eventual conclusion of this project? Is there a way we can prepare for that transition together? |
| --- | --- |
| Training and Skill Development | - Reflecting on the initial training sessions, how helpful were the series of trainings on research methods and related topics in preparing you for the work? - Were there any topics not covered in the trainings that you would have liked to receive training on? - Do you have any feedback or suggestions regarding the structure or content of the trainings we conducted? - Are there any specific skills or experiences you hope to gain before the project concludes? |
| Technology and Tools | - What has your experience been like using unfamiliar technology or software as part of this project? - Would additional training on tools such as Zoom or Windows be helpful for future work? |
| Work Environment and Logistics | - What are your thoughts on the hybrid work model (combining in-person and online participation)? Were there any benefits or challenges? - What are your impressions of the Hans Rosling Center as our in-person meeting location? What aspects were helpful or challenging? |
| Compensation and Support | - For those who received a stipend to cover expenses such as transportation, how did you feel about receiving it as a direct payment versus having specific costs covered (e.g., bus vouchers)? - Do you think it would be helpful to individualize compensation methods based on personal preferences or needs? |
| Impact and Future Directions | - What changes would you like to see as a result of the work we are doing? - Do you believe the work we are doing is likely to make a meaningful difference? - Are there any future goals, aspirations, or directions you would like to see the group pursue? |

Supplementary Material 4. New project/contract request form with criteria for evaluating new projects

| **Criterion** | **Question** |
| --- | --- |
| Type of organization | Please describe your organization and your role there. |
| Scope of project | Please describe the proposed project in as much detail as possible |
| Available budget | What is the projected budget available for partnership with READU? |
| Proposed role | How do you envision READU's role in this project?  Please describe in as much detail as possible. |
| Consultation history | Please describe your group's history with consulting people who use drugs (PWUD) |
| Potential impact | How will this project positively impact health equity for people who use drugs? |
| Shared values | What does meaningful engagement with PWUD mean to you? |
| Available time | What is the timeline for this project? Keep in mind that READU's process for doing research may take longer than other  groups. |
